# On the Origins of Enzyme Inhibitor Selectivity and Promiscuity: A Case Study of Protein Kinase Binding to Staurosporine

**DOI:** 10.1111/j.1747-0285.2009.00832.x

**Published:** 2009-07

**Authors:** Duangrudee Tanramluk, Adrian Schreyer, William R Pitt, Tom L Blundell

**Affiliations:** 1 Department of Biochemistry, University of Cambridge80 Tennis Court Road, Cambridge CB2 1GA, UK; 2 UCB Celltech208 Bath Road, Slough, Berkshire, SL1 3WE, UK

**Keywords:** ATP-binding site, kinase selectivity, Mantel test, staurosporine, structural analysis

## Abstract

Relationships between ligand binding and the shapes of the binding sites in families of homologous enzymes are investigated by comparing matrices of distances between key binding site atoms. Multiple linear regression is used to help identify key distances that influence ligand binding affinity. In order to illustrate the utility of this generic approach, we study protein kinase binding sites for ATP and the promiscuous competitive inhibitor, staurosporine. We show that the size of the gatekeeper residue and the closure between the first glycine of the GXGXXG motif and the aspartate of the DFG loop act together to promote tight binding. Our web-based tool, ‘mapping analogous hetero-atoms onto residue interactions’ (MAHORI), indicates that the greater the number of hydrogen bonds made by the kinase around the methylamine group of staurosporine, the tighter the binding. The conservation of surrounding atoms identified using our novel grid-based method clearly demonstrates that the most structurally conserved part of the binding site for staurosporine is the main chain of the hinge region. The critical role of interactions that are not dependent on side-chain identities is consistent with the promiscuous nature of this inhibitor.

Protein kinases catalyze the transfer of a phosphate group, usually from ATP, to a protein substrate. The human genome comprises more than 500 protein kinases (1), which are known to mediate most of signal transduction crucial to metabolism, cell proliferation and differentiation, membrane transport, and apoptosis. Although they exhibit a variety of regulatory mechanisms and conformational states, protein kinases share the same fold and similar ATP-binding sites (2).

Protein kinases are of considerable interest to the pharmaceutical industry because dysfunction often results in malignancy (3,4). Kinases are also validated anti-inflammatory drug targets (5). Indeed, the success of several high-affinity ATP-mimetic drugs has made the design of selective inhibitors an attractive approach to useful therapeutics, particularly for oncology (6). Although the structural conservation of the ATP-binding site can lead to off-target ligand binding, kinase inhibitor design has become a promising way forward for discovery of useful therapeutic agents (7).

The major challenge for protein kinase inhibitor design is obtaining selectivity. In order to reduce the chances of undesirable side-effects, potency is usually optimized against a target kinase while reducing off-target activities including those involving homologous proteins. However, the level of selectivity required is dependent on the therapeutic endpoint. Indeed, in some cases, polypharmacology is an advantage, although a multi-target selectivity is very difficult to achieve by design (8).

Insights into protein kinase selectivity and promiscuity are the major objectives of this article. Although high-affinity targets sometimes have similar residues at positions important for binding of a given kinase inhibitor, others with similar residues at these important positions can be insensitive to such inhibitors, probably because of conformational differences (9). Therefore, understanding kinase selectivity cannot be achieved only through the analysis of sequences but must also consider three-dimensional structures.

Here, in order to understand the relationship between the protein structure and binding affinities, we study complexes of protein kinases with staurosporine (10), a microbial alkaloid isolated from *Streptomyces staurosporeus*. Although staurosporine is a potent inhibitor of various human protein kinases and has some antifungal activity, it is too toxic to be used as a drug. However, it has been widely used in research as a universal kinase inhibitor (11). The three-dimensional structures of a significant number of protein kinases co-crystallized with staurosporine can be found in the Protein Data Bank. They show that staurosporine mimics ATP very well, in spite of the apparent lack of similarity between the two molecules. The availability of dissociation constants (*K*_d_) for staurosporine with 119 kinases (12), when used in combination with this structural information, allows us to relate binding affinities to differences in the structures of the pockets.

We have studied the relationship between the structures of protein kinases and their ability to bind promiscuous ligands, which we define here as compounds that bind several and, in the case of staurosporine, the majority of kinases on which they are tested. In order to investigate the factors that determine whether a particular kinase will bind a particular kinase inhibitor, we have examined the regions around the pockets of a set of medium-to-high-quality X-ray crystal structures; and to group kinases based on the similarity in spatial arrangement of amino acid side chains, we use the Mantel test, a statistically robust method for calculating correlation coefficients between distance matrices (13). Our shape-based dendrogram shows that similarity in shape alone can sometimes determine the affinity for a set of ligands, regardless of their overall sequence similarity.

The study of the shapes of the pockets allows us to identify the factors required for most kinases to bind the promiscuous inhibitor, staurosporine. We examine the similarities of the shapes of the pockets by identifying neighboring entities that are conserved in their positions relative to staurosporine. We show that some are recruited to staurosporine via an induced fit or conformational selection mechanism, which contributes to staurosporine promiscuity. We focus on the differences in distance matrices that define the pockets and show how the side chains affect binding affinities. Based on a quantitative structure activity relationship (QSAR) approach, we select a set of distances that have an influence on binding affinities. These distances indicate that tighter binding is associated with the closure of the N-lobe and C-lobe and a larger size of the gatekeeper residue. The numbers of ionic interactions and hydrogen bonds around the methylamine of staurosporine also affect the binding affinities.

## Methods

### Shape correlation by Mantel test

Protein Data Bank identification codes of structures with ‘protein kinase activity’, i.e., gene ontology ID GO:0004672 on MSDlite database (14), were filtered through the PISCES server in order to select representative protein crystal structures based on resolution, R-factor and completeness (15). The 80 chosen structures with resolutions better than 3.0 Å and R-factors less than 0.30 were then superposed onto cyclic AMP-dependent protein kinase (PKA) using the program baton based on the method developed in Comparer (16). PKA, the first protein kinase for which a crystal structure became available (17), was selected as the template because its residue nomenclature is widely used in kinase analyses (18). The obtained structural alignment was used to infer equivalent residues in the kinase superfamily. Distances between every residue surrounding the pocket were calculated. This was achieved by selecting a ‘representative’ atom, generally located near the end of the side chain for each amino acid residue. For example, the hydroxyl oxygen was chosen for serine and the beta-carbon was chosen for threonine (see Appendix S2). Half-diagonal distance matrices were constructed and the correlations between the matrices were calculated by the Mantel test using program zt (19). Relationships between distance matrices were defined using the neighbor-joining algorithm from the program phylip (20) and the dendrogram was made for comparison using the program treeview (21). Inter-residue distances were used which characterize a particular kinase regardless of the ligand with which it was crystallized. The equivalent set of distances was also measured for another set of 35 non-redundant crystal structures of kinases, which have been assayed by Fabian *et al.* (12). These structures contain a variety of inhibitors bound in the ATP-binding site. This allows the relationship between the spatial arrangement of residues of different kinases and their binding affinities (*K*_d_) against 10 ligands to be visualized. We used the color gradient representation of the tree plotting program itol (22), where the intensities of the colors were proportional to −log_10_*K*_d_, in order to allow comparison for millimolar to sub-nanomolar values of binding constants (*K*_d_).

### Position-specific interactions

The structures of 20 staurosporine–kinase complexes were superposed on the indolocarbazole moiety of staurosporine in PDB ID 1stc in order to compare them with the structures of 24 adenosine phosphate–kinase complexes, which were superposed on the adenine ring from ATP in PDB ID 1atp. The position-specific interactions were considered at two levels: the atom type and the residue type. For the atom-based arrays, atoms in the PDB file were assigned an atom type according to the simplified approach used in the AMBER force field, which has been developed specially for molecular mechanics calculations of proteins and nucleic acids (23) (Appendix S1). For the residue-based arrays, only ‘representative’ atoms and oxygen of water molecules were assigned a residue type (see Appendix S2). We constructed a three-dimensional grid with 1 Å dimensions around the rigid part of the superposed ligands and collected occupancy in the grid boxes based on residue or atom types and x, y, and z coordinates for non-redundant protein kinase structures. For the atomic level, the grid was stored in PDB file format and occupancies of boxes were contoured using the module color_b of program pymol (24) to obtain a transparent surface with the intensity of the color corresponding to the frequency with which the grid boxes are populated. For the residue level, relative positions of neighboring residues for different ligands were observed by superposing the majority of the residue clusters surrounding adenine and staurosporine, and then comparing the positions of these clusters for adenine complexes and staurosporine complexes. For visualizing the cluster, a bond is drawn automatically when two atoms come closer than about 2 Å so the cluster can be easily found. Colors of frequently occurring residues are assigned according to the type of amino acid found in the array.

### Distance-based QSAR

We filtered the PDB (25) for X-ray structures of the kinases for which Fabian *et al.* (12) report a *K*_d_ for staurosporine (*K*_d,STU_), and selected only those structures that are co-crystallized as either staurosporine or adenosine phosphate complexes (40 structures). As in the case of shape analysis, distances from representative atoms between 15 residues surrounding the pocket were measured to find the best correlation of distances with *K*_d,STU_. Multiple linear regression was performed using the program xlstat (26) to find the best equation relating the distances measured between the centre points near the ends of the side chains (see Appendix S2) and log_10_*K*_d,STU_. Amino acid residues used to create these distance descriptors were extracted by referring to PKA equivalent residues from the ClustalX (27) multiple sequence alignment of the 113 kinases in Fabian’s data set (12). The relationships of these influential residues were drawn from the neighbor-joining algorithm in ClustalX (27). The abilities of kinases to bind the inhibitors staurosporine, LY-333531, SU11248, and ZD-6474 were calculated from −log_10_*K*_d,_ and a dendrogram was produced with gradient color representation using program itol (22) in order to reflect these values. The selected inhibitors are among the most promiscuous ligands in the Fabian data set, so that the number of the kinases they can bind is sufficient to demonstrate trends in binding affinities.

Because the experimental data depend not only on the method used but also the experimentalist who reports the values, we choose dissociation constants of staurosporine (*K*_d,STU_) from Fabian *et al.* (12) as the sole source of our experimental binding data. Structures in this training set structure have *K*_d,STU_ between 0.5 and 870 nm and both adenine-containing and staurosporine-bound structures are considered. We include adenine ring-containing structures in the data set on the assumption that the rigid part of the pockets that harbor adenosine or staurosporine share similar conformations and electronic features. The advantage of assuming that the structure of adenosine phosphate-bound complex resembles that of the same enzyme in the staurosporine-bound complex is that there are many structures in complex with adenosine containing compounds. The greater number of structures with available *K*_d,STU_ allowed us then to test our equation by predicting *K*_d,STU_ for further kinase structures co-crystallized with adenine ring-containing ligands. We discarded residue points that differ in position when found in contact with ATP or staurosporine, so that we could be sure that the differences in distances were independent of the ligand bound.

### Chemical interactions of staurosporine with various kinases

MAHORI (Mapping analogous hetero-atoms onto residue interactions) is our web-based tool designed to observe interactions surrounding specific parts of a ligand^a^. The website allows for various types of ligand query e.g., chemical drawing, compound name, PDB ligand three-letter code and SMILES string. Given the PDB ligand three-letter hetID of staurosporine (STU), MAHORI searches the PDB for the staurosporine complexes against the whole PDB using the PDB-ligand database CREDO (28). This database stores protein–ligand interactions using criteria adapted from Marcou and Rognan (29). All atoms from the ligand and their contacting neighbor atoms from the protein have predefined types. Every contacting atom pair is assigned an interaction type by considering the type, the geometry and the threshold distance. The details of residues that interact with the queried atoms are presented according to the type of interaction, so that molecular interactions of multiple structures can be compared at the level of ligand substructure.

## Results and Discussion

### Ability to bind inhibitors correlates with spatial arrangement of residues in the pocket

**Figure 1:**
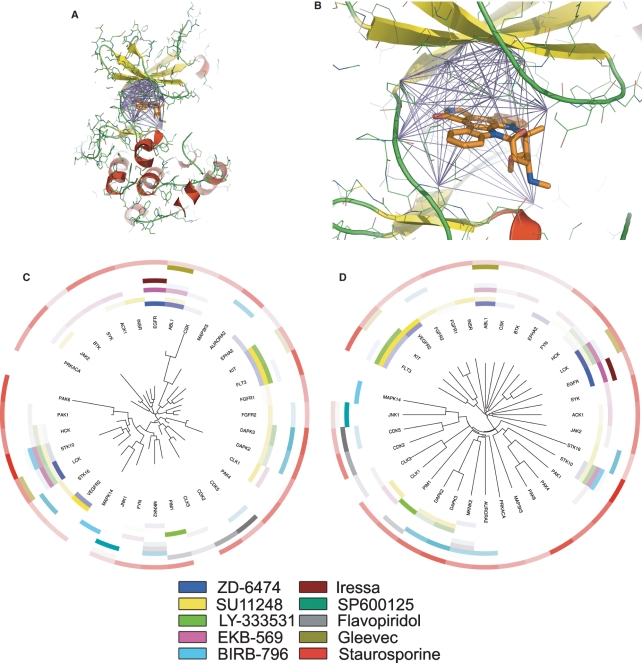
Correlation between the shapes of the kinase ATP-binding pockets and their binding affinities to inhibitors. (A, B) The quasi-shape of the adenine-binding pocket of cAMP-dependent protein kinase (PDB ID:1stc, chain E) in complex with staurosporine (orange stick) is illustrated by drawing purple lines between the ‘representative’ atoms of the 17 residues surrounding the pocket. (C) The shape-based dendrogram shows the matrix correlation between the shapes of the kinases and their binding affinities to 10 inhibitors. The method employs 17 active-site residues to construct the distance matrices for each kinase and then find the correlations between them. The pocket of STK10, an STE kinase, shows the greatest similarity in shape to that of LCK, a tyrosine kinase. Their inhibition profiles appear very similar (lower left). (D) The classic dendrogram based on sequence similarity from structure-based sequence alignment using program baton. The intensity of the color is proportional to log_10_*K*d of the inhibitor. It clusters the kinase with similar performance to the shape-based dendrogram. Staurosporine is the most promiscuous inhibitor in this data set (red).

We wished to investigate whether the spatial arrangement of residues in the ATP-binding pocket has an influence on which inhibitor the kinase recognizes. In order to avoid comparing extremely variable regions of the pocket, we focused only on protein structures with staurosporine or adenine ring-containing compounds bound. We selected a set of points to represent common features of the pocket and used the Mantel test to distinguish the pockets of different kinases based on the assumption that the matrix of distances between points surrounding the adenosine pocket can reflect key features of the pocket shape in multi-dimensions; we call a matrix of this sort a ‘quasi-shape’ (Figure 1A and 1B). We used calculated correlation coefficients among distance matrices of the same size and order of elements to estimate the similarities in the spatial arrangements of side chains and hence the relationships between shape and the ability of parent kinases to bind various inhibitors.

Our preliminary results suggest that Mantel test correlations between the matrices derived from a small set of inter-atomic distances can separate the majority of staurosporine complexes from the adenine-containing complexes. This means that there are observable differences in spatial arrangement of these atoms when staurosporine and adenine are bound. Thus, the Mantel Test appears to work well for classifying different three-dimensional geometric shapes. However, the same kinase in different crystal forms can be scattered throughout the resulting shape-based dendrogram, implying that similarities between conserved atoms are not able to identify identical kinases in different conformational states. Therefore, we investigated the use of ‘representative’ atoms as the centers for distance measurements in the construction of the distance matrix, thus allowing the derivation of a quasi-shape from each PDB file. The choice of these atoms, which depends on their residue type, is shown in Appendix S2. By gradually increasing the number of residue points, we were able to cluster the same kinase in different crystal forms and different complexes into the same branch of the dendrogram. The most useful shape-based dendrogram is constructed from 17 representative points from 17 residues. These are equivalent to the following residues in PKA: LEU 49, GLY 50, VAL 57, ALA 70, MET 71, LYS 72, VAL 104, MET 120, GLU 121, TYR 122, VAL 123, GLU 170, ASN 171, THR 183, ASP 184, GLU 127, LEU 173. This dendrogram places the same type of kinase in different complexes in the same branch regardless of the bound ligand, and the staurosporine binding structures were clustered into one-half of the tree (Appendix S3).

We then applied this method to a set of 35 non-redundant protein kinases and 10 inhibitors from the Fabian *et al.* data set (12) and obtained a dendrogram that characterizes the ability to bind 10 ligands, based on the similarity in quasi-shape (Figure 1C). The general sequence-based dendrogram is shown for comparison (Figure 1D). It is evident that kinases with similar pocket quasi-shapes are likely to have similar inhibitor binding profiles, regardless of their family membership. A nice example is serine/threonine kinase 10 (STK10), which is clustered in the sequence-based dendrogram as an STE kinase, or Homolog of yeast Sterile 7, 11, 20 kinases, as defined in the protein kinase phylogenetic tree by Manning *et al.* (1). When considering STK10 in terms of similarity in spatial arrangement of residues, it is instead paired with leukocyte-specific protein tyrosine kinase (LCK) which is a tyrosine kinase. The sequences are quite different, but the quasi-shape of the pockets and their abilities to bind seven inhibitors are very similar. Many kinases with similar sequences, for example CDK2 and CDK5 or DAPK2 and DAPK3, also have very similar quasi-shapes and inhibition profiles. This quasi-shape-based dendrogram provides a way of visualizing relationships among kinases, complementing that of the classical sequence-based dendrogram. Our dendrogram demonstrates that the similarity in quasi-shape can sometimes explain the ability to bind a set of ligands regardless of the overall sequence identity.

### Conserved interaction shows induced-fit mechanism upon staurosporine binding

We hypothesize that if protein features surrounding a particular ligand remain conserved both in atom type and position in complexes of different kinases, they may be required for binding the ligand. We have developed software to extract generalized features that are frequently found in protein kinase structures by constructing four-dimensional arrays to capture different entities that are conserved in atomic position on structure superposition of staurosporine complexes. The array collects occupancies of atoms from superposed structures that satisfy the four criteria, i.e., x,y,z coordinates and atom type. This approach increases signal to noise by superposing a significant number of structures (20 structures of staurosporine complexes).

**Figure 2:**
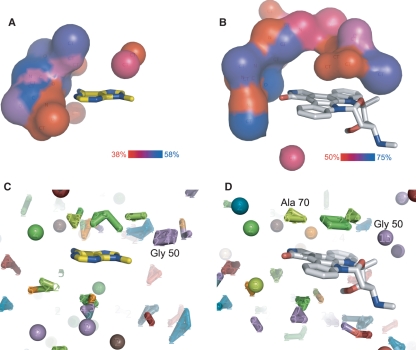
Frequently occurring atoms and residues around the ATP- and staurosporine-binding sites. The hinge region is on the left, the N-terminal lobe at the top and the C-terminal lobe at the bottom of the figure. (A,B) Color is related to the frequency of finding atoms at a position (CT = sp^3^ carbon, C = carbonyl sp^2^ carbon, N = sp^2^ amide nitrogen, O = sp^2^ oxygen). Both staurosporine (white stick) and the adenine ring (yellow stick) are recognized by the main chain atoms in the hinge region on the left. The atomic environment of adenine (A) shows more variability than that of staurosporine (B) as shown by the maximum occupancy of frequently occurring atoms (58%); this is smaller than that of staurosporine (75%). (C) The first glycine of the GXGXXG motif in purple moves within a rhombohedron-shaped volume when located near the adenine ring. This glycine retains its position in the same grid in 75% of staurosporine structures (15 from 20 structures) indicating induced fit of the glycine-rich loop when located near staurosporine (D).

The staurosporine molecule is quite rigid as it contains very few rotatable bonds; hence, we may observe interaction partners that are position-specific by superposing the kinases onto its lactam and indolocarbazole rings. In a similar manner, interactions around the adenosine phosphate complexes can be compared by superposing the non-redundant kinase structures onto the adenine ring (24 structures of adenine-containing complexes). The conserved atomic environment can be found by observing the frequently occurring atoms at a particular location defined by a 1 Å grid box (Figure 2A and 2B).

The atom type categorization is based on the assumption that atoms around the side chains that have the same functional group can be classified as the same atom type, e.g., carboxylate oxygens of Asp and Glu have sp^2^-oxygen atom types (Appendix S1). By this approach, we can capture similar interactions in the pocket made by similar parts of ligands. The resulting atomic level arrays suggest that for both adenine and staurosporine ligand complexes (Figure 2A and 2B), the main chain atoms of the kinase make the most conserved interactions in terms of type and position, which explains why staurosporine, mimicking ATP, can bind to most of the kinases.

The conserved neighboring atoms of ATP are more variable in position than those of staurosporine (38–58% conservation in Figure 2A versus 50–75% in Figure 2B). This supports the idea that the two ligands require a different degree of flexibility within the active site of the kinase. The greater number of rotatable bonds in the adenosine phosphate results in lower degree of conservation of neighboring atoms in these complexes than in staurosporine complexes. The ribose moiety of the adenosine complex can adopt several conformations, so the frequently occurring atoms fall in several grid boxes. The main chains of protein kinases in the hinge regions interact with the amine group of the adenine rings and are the most conserved parts of the adenine complexes. In a similar way, the main chains of the hinge regions, which interact with the lactam oxygen, and the α-carbons of the first glycines of the GXGXXG motifs, which interact with the tetrahydropyran, are the most conserved parts for staurosporine complexes. Furthermore, for 15 out of 20 staurosporine-bound structures, the main chain alpha carbons from the first glycine of the G-rich loop fall in the same 1 Å grid box (Figure 2D). When superposed on the adenine ring, these atoms are distributed within a rhombohedron-shaped volume (see Figure 2C). It is perhaps surprising that this glycine can become fixed in position upon staurosporine binding because the glycine-rich loop is generally believed to be highly flexible (30,31). The relatively well-conserved position of this glycine, but not for adenine complexes, suggests that the staurosporine causes an induced fit or conformational selection in the kinases upon binding. However, further evidence from apo structures is required to confirm this observation.

The residue arrays serve to complement the pictorial representations of the atomic environment. For most of the residues, we chose the penultimate atoms of the side chains to represent the identities and the positions of the residues (Appendix S2). In this way, residues with a similar functional group at the end of the side chain in different kinases can be captured as points at similar locations in the superposed structures. For instance, C_β_ of valine and C_γ_ of leucine at the active sites occupy the same or close-by grid boxes in the superposed structures. We observe that some clusters of amino acids preserve their functional groups in most of the staurosporine and the adenine complexes; examples are the side chains of glutamate and aspartate of the salt-bridges which flank the pocket on both sides, the residues that are equivalent to Ala_70_ in PKA which acts as the ceiling of the cleft, and again Gly_50_ which interacts with the ether oxygen of the ribose (Figure 2C and 2D). Several clusters of amino acids are seen to have moved, leading to contraction and expansion of the residues in the pocket to accommodate staurosporine.

The ends of some similar hydrophobic side chains, including Cys and Met, or Ala, Val and Leu, which surround the planar indolocarbazole ring, have equivalent positions, implying that these amino acids perform the same function in that part of the active site. On the contrary, the amino acids that make contact around the methyl amino and methoxy groups of staurosporine demonstrate that remarkably different functional groups can occupy the same position and carry out the same structural role.

### Distance descriptors and interaction types correlate with the magnitudes of binding affinities

Staurosporine has very few strongly electrostatic features. Its major interactions with protein kinases are largely steric with non-polar groups. Thus, we hypothesized that the tightness of inhibitor binding might be determined by the compactness of residues in the pocket. In order to test this idea and to predict binding affinities from the structures, we assumed that good binding requires certain geometric restraints and investigated which distance descriptors correlate well with the dissociation constants. Thus, if distances are shorter for most of the structures with low binding constants, we investigate whether contraction along that direction is required for tight binding. This approach resembles QSAR, but all the input parameters are measured from the structure in terms of distances that constitute the quasi-shape of the ATP-binding pocket. Although QSAR methodologies have been widely used to try to understand binding affinities through various parameters related to lipophilicity, charge and hydrogen bonding character (32), distances between certain atoms in the protein have not been used.

In order to select distances from the quasi-shape defined by 15 points in contact with staurosporine (Figure 1A and 1B; Appendix S4), we carried out multiple linear regression with the equations shown in Table 1. We tested the predictive power of these equations by leaving out randomly selected test sets. While the purpose of using multiple linear regression in this context was simply to select the set of distances that correlate well with the binding affinities, the resulting equations suggest that predictive power might be demonstrated if a larger data set were available. All resulting equations appear to contain the same best sets of distances producing *R*^ 2^ values for the random test sets of about 0.7 for both equations (Appendix S5).

**Table 1:** Equations correlating the influential distances with log_10_*K*_d,STU_

Random Test	*R* ^2^ training	*R* ^2^ test set	Equation
None	0.6	–	log_10_*K*_d,STU_ = 3.4 + 0.1D_50_184_−0.4D_120_123_
5 Structures	0.6	0.7	log_10_*K*_d,STU_ = 3.4 + 0.1D_50_184_−0.4D_120_123_
10 Structures	0.7	0.7	log_10_*K*_d,STU_ = 3.6 + 0.1D_50_184_−0.4D_120_123_

**Figure 3:**
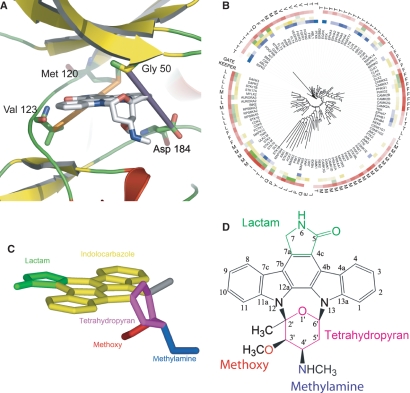
The interpretation of multiple linear regression equations. (A) Interpretation of the multiple linear regression analysis shows that smaller values of *K*_d,STU_ result from the larger size of side chains of the gatekeeper and gatekeeper + 3 residues, i.e., PKA equivalent residue: Met_120_ and Val_123_ (orange bar). The equation suggests that the closer approach between Gly_50_ of the N-terminal lobe and Asp_184_ of the C-terminal lobe (purple bar) correlate with tighter binding to staurosporine. (B) A dendrogram displaying relationships between 113 kinases based on neighbor-joining of the 13 residues which are in contact with staurosporine and show correlation (<−0.4 and >0.4) with *K*_d,STU_. The aim is to investigate whether the similarities between these influential residues, equivalent to PKA residues 49, 50, 57, 70, 71, 72, 120, 121, 122, 123, 170, 171, and 184, give rise to similar binding constants. The resulting dendrogram can cluster staurosporine tight binders into two major groups with better binding affinity to staurosporine (dark red). This group of kinases tends to have large gatekeeper residues, e.g., Phe (F), Met (M). Smaller gatekeeper residues, e.g., Thr or Leu, tend to be associated with weaker binding affinities to staurosporine. A majority of kinases which are inhibited by ZD-6474 (blue) has threonine (T) or valine (V) as a gatekeeper residue. Binding affinities to LY-333531 (green) and SU11248 (yellow) are shown for comparison. (C) Staurosporine structural components. (D) Chemical structure of staurosporine based on annotation from Zhao *et al.* (33).

The distance descriptors which correlate well with binding affinities, either having positive or negative influence on *K*_d,STU_, are called the ‘influential distances’. Although correlation does not imply causation, examination of the crystal structures allows us to interpret these results in terms of structure. Figure 3A illustrates these influential distances in the structure of PKA, PDB ID 1stc. They can be used to describe how positions of representative side-chain atoms can influence *K*_d,STU_ and also to help understand the major changes in neighboring atomic positions around the staurosporine (Figure 3C).

Of the equations shown in Table 1, the distance between residues 50 and 184, described in equation as D_50_184_, is directly proportional to the value of log_10_*K*_d,STU_, and the distance between residue 120 and 123, D_120_123_, is inversely proportional to log_10_*K*_d,STU_. A possible interpretation of the equation is that in kinases that are tightly bound to staurosporine, i.e., have a small log_10_*K*_d,STU_, there is a preference for a smaller D_50_184_ and a larger D_120_123_. In PKA, the distance between residues 50 and 184 is measured between Cα of Gly_50_ of the GXGXXG motif in N-terminal lobe to Cγ of Asp_184_ of the DFG loop in C-terminal lobe. Staurosporine is located between the two lobes, and the closer approach of these two motifs in a direction perpendicular to the plane of staurosporine reflects the better binding affinities presumably because of the resultant tighter binding. In contrast, the distance between residue 120 (gatekeeper) and 123 (gatekeeper + 3) implies the expansion of the pocket along this direction. The equation suggests that these two residues should move further apart to accommodate staurosporine. The gatekeeper residue points toward the plane of staurosporine, while the gatekeeper + 3 residue is located under the indolocarbazole ring. The size of the gatekeeper and the gatekeeper + 3 residues appear to have a key role in locking the lactam in the correct orientation while making optimal steric interactions with the indolocarbazole of staurosporine. The larger size of the gatekeeper residue likely results in the larger distance and correlates with good binding because the larger volumes of the side chains in the plane of the lactam ring promote favorable hydrophobic interactions in the pocket.

We speculated that the interactions involving the methyl amino (N_4′_) and the methoxy group (O_3′_) of staurosporine should constrain the distance between the N- and C-terminal lobes to the optimal value (Figure 3D). Indeed, the hydrogen bonds or ionic interactions that the staurosporine can make along this direction are associated with the major differences in the binding affinities. We find that the number of hydrogen bonds made by residues around N_4′_ of staurosporine corresponds well with the trend in binding affinities (Table 2). Kinase structures that have two residues making hydrogen bonds or ionic interactions to N_4′_ of staurosporine, i.e., CDK2, PKA, PIM1, and LCK, have binding affinities below 51 nm. Most structures that have only one residue contributing hydrogen bonds or ionic interaction to N_4′_ have binding affinities between 51 and 440 nm, i.e., CSK, EGFR, FYN, M3K5. The kinase STK16 which does not make any interaction with N_4′_ has a binding affinity of 200 nm.

**Table 2:** Number of interactions made by the kinases with N_4′_ of staurosporine

			Number of interactions		
Protein kinase	PDB ID	*K*_d,STU_ (nm)	H-bond	Ionic	vdW
CDK2	1AQ1	8.1	2	1	2
PIM1	1YHS	15	2	1	2
LCK	1QPJ	20	2	–	2
PKA	1STC	50	1	1	2
KSYK	1XBC	7	1	–	1
FYN	2DQ7	51	1	–	1
M3K5	2CLQ	120	1	–	1
CSK	1BYG	440	1	–	1
MKNK2	2HW7	22	–	–	1
EGFR	2ITU	70	–	1	2
STK16	2BUJ	200	–	–	–

Therefore, in order to modify staurosporine to achieve better affinity for kinases, the strategy might be to identify a residue close to the methyl amino (N_4′_) and to modify the staurosporine to make another hydrogen bond. Making point mutations of active site residues in order to achieve a better binding affinity to staurosporine might also be achieved by selecting the residue type that can make an optimal hydrogen bond to N_4′_ and O_3′_ of staurosporine.

## Conclusion

Simple comparison of distance matrices, generated from representative atoms toward the ends of side-chains, can be used to describe the geometry of a ligand binding site and this can be related to inhibitor binding. By considering the similarities and differences in the active sites, our computational approach can rediscover several kinase binding determinants that have been previously identified from manual and experimental analyses. We show by grouping structures with similar inhibition profiles that the shape of the pocket can contribute to inhibitor selectivity. The most significant part of the protein kinase structures that remains fixed in type and position for both staurosporine and adenosine structures is the main chain at the beginning of the hinge region. This implies that the reason that staurosporine binds to most kinases is that the lactam from staurosporine and adenine from ATP recognize a similar set of atoms. Our observation gives a more precise picture of the induced fit of the conserved glycine-rich loop upon binding to staurosporine. In addition, our statistical analysis shows that a larger size of the gatekeeper residues normally results in tight binding to staurosporine. We have also learned that the hydrogen bond and ionic interaction made with methylamine is important to the tightness of the binding with staurosporine.

These results indicate that by understanding differences in the active sites, we can identify residues that affect the ability to bind the inhibitor and also suggest the part of the inhibitor that might be modified to achieve better binding affinities. This approach offers a new perspective on computational descriptions of specificity determinants when a series of different proteins with the same ligand becomes available.

## Footnotes

a http://www-cryst.bioc.cam.ac.uk/mahori

## Supporting Information

**Appendix S1.** Classification of atom types in amino acids.

**Appendix S2.** Reference atoms for the classification of amino acids at the residue type level.

**Appendix S3.** The shape-based dendrogram constructed from 17 points in 17 residues.

**Appendix S4.** Distances between pairs of 15 frequently occurring residues chosen for Multiple Linear Regression.

**Appendix S5.** The predictive power of the multiple linear regression equations, tested by leaving out randomly selected test sets.
