# Supplementary material for: On the Origins of Enzyme Inhibitor Selectivity and Promiscuity: A Case Study of Protein Kinase Binding to Staurosporine
Source: Chem Biol Drug Des. 2009 Jul;74(1):16–24. doi: 10.1111/j.1747-0285.2009.00832.x (PMC2737611; doi:10.1111/j.1747-0285.2009.00832.x)
Supplement: Supplementary file 1 [file jpp0074-0016-SD1.doc]

**Appendix S1** Classification of atom types in amino acids

The following atom types are assigned to all atoms in the grid-box according to the element type.

#### Carbons

1. **CT**: any sp^3^ carbon
2. **C**: any carbonyl sp^2^ carbon
3. **CA**: any aromatic sp^2^ carbon and C_ε_ of Arg
4. **CH**: all of histidine’s aromatic carbons except for CD2 (*i.e.* sp^2^ aromatic carbon in 5-membered ring with one substituent and next to nitrogen, or next to carbon and lone pair nitrogen, or next to two nitrogens)
5. **CW**: tryptophan’s carbon in connection with the 5-membered ring (*i.e.* sp^2^ aromatic in 5-membered ring next to carbon and NH, or at junction of 5- and 6-membered rings, or next to two carbons, or sp^2^ junction between 5- and 6-membered rings and bonded to CH and NH)
6. **CX**: any other unidentified carbon including carbon from the bound ligand

**Nitrogens**

1. **N** : sp^2^ nitrogen in amides
2. **NC** : sp^2^ nitrogen in aromatic rings (sp^2^ nitrogen with hydrogen attached, sp^2^ nitrogen in 5-membered or 6-membered ring with lone pair electrons)
3. **N2** : sp^2^ nitrogen of aromatic amines
4. **N3** : sp^3^ nitrogen
5. **NX**: any other unidentified nitrogen including nitrogen from the bound ligand

**Oxygens**

1. **OH** : sp^3^ oxygen in alcohols, tyrosine, and protonated carboxylic acids
2. **O**: sp^2^ oxygen in amides
3. **O2** : sp^2^ oxygen in anionic acids
4. **OW**: oxygen in water
5. **OX**: any other unidentified oxygen

**Sulfur**

1. **SH**: cysteine sulphur
2. **S**: methionine sulphur
3. **SX**: any other unidentified sulphur

**Phosphorous**

1. **P**: phosphorus in phosphates

**Halogens**

1. **X**: F, Cl, Br, I

| **Atom** |  |  | **Type** | **Atom** |  |  | **Type** | **Atom** |  |  | **Type** |
| --- | --- | --- | --- | --- | --- | --- | --- | --- | --- | --- | --- |
| Glycine | N |  | N | Aspartate | N |  | N | Histidine | N |  | N |
| Glycine | CA |  | CT | Aspartate | CA |  | CT | Histidine | CA |  | CT |
| Glycine | C |  | C | Aspartate | C |  | C | Histidine | C |  | C |
| Glycine | O |  | O | Aspartate | O |  | O | Histidine | O |  | O |
| Alanine | N |  | N | Aspartate | CB |  | CT | Histidine | CB |  | CT |
| Alanine | CA |  | CT | Aspartate | CG |  | C | Histidine | CG |  | CH |
| Alanine | C |  | C | Aspartate | OD |  | O2 | Histidine | ND1 |  | NC |
| Alanine | O |  | O | Asparagine | N |  | N | Histidine (HE/+) | CD2 |  | CW |
| Alanine | CB |  | CT | Asparagine | CA |  | CT | Histidine (HD) | CD2 |  | CH |
| Valine | N |  | N | Asparagine | C |  | C | Histidine | CE1 |  | CH |
| Valine | CA |  | CT | Asparagine | O |  | O | Histidine | NE2 |  | NC |
| Valine | C |  | C | Asparagine | CB |  | CT | Phenylalanine | N |  | N |
| Valine | O |  | O | Asparagine | CG |  | C | Phenylalanine | CA |  | CT |
| Valine | CB |  | CT | Asparagine | OD1 |  | O | Phenylalanine | C |  | C |
| Valine | CG |  | CT | Asparagine | ND2 |  | N | Phenylalanine | O |  | O |
| Leucine | N |  | N | Glutamate | N |  | N | Phenylalanine | CB |  | CT |
| Leucine | CA |  | CT | Glutamate | CA |  | CT | Phenylalanine | CG |  | CA |
| Leucine | C |  | C | Glutamate | C |  | C | Phenylalanine | CD |  | CA |
| Leucine | O |  | O | Glutamate | O |  | O | Phenylalanine | CE |  | CA |
| Leucine | CB |  | CT | Glutamate | CB |  | CT | Phenylalanine | CZ |  | CA |
| Leucine | CG |  | CT | Glutamate | CG |  | CT | Tyrosine | N |  | N |
| Leucine | CD |  | CT | Glutamate | CD |  | C | Tyrosine | CA |  | CT |
| Isoleucine | N |  | N | Glutamate | OE |  | O2 | Tyrosine | C |  | C |
| Isoleucine | CA |  | CT | Glutamine | N |  | N | Tyrosine | O |  | O |
| Isoleucine | C |  | C | Glutamine | CA |  | CT | Tyrosine | CB |  | CT |
| Isoleucine | O |  | O | Glutamine | C |  | C | Tyrosine | CG |  | CA |
| Isoleucine | CB |  | CT | Glutamine | O |  | O | Tyrosine | CD |  | CA |
| Isoleucine | CG1 |  | CT | Glutamine | CB |  | CT | Tyrosine | CE |  | CA |
| Isoleucine | CG2 |  | CT | Glutamine | CG |  | CT | Tyrosine | CZ |  | C |
| Isoleucine | CD |  | CT | Glutamine | CD |  | C | Tyrosine | OH |  | OH |
| Serine | N |  | N | Glutamine | OE1 |  | O | Tryptophan | N |  | N |
| Serine | CA |  | CT | Glutamine | NE2 |  | N | Tryptophan | CA |  | CT |
| Serine | C |  | C | Methionine | N |  | N | Tryptophan | C |  | C |
| Serine | O |  | O | Methionine | CA |  | CT | Tryptophan | O |  | O |
| Serine | CB |  | CT | Methionine | C |  | C | Tryptophan | CB |  | CT |
| Serine | OG |  | OH | Methionine | O |  | O | Tryptophan | CG |  | CW |
| Threonine | N |  | N | Methionine | CB |  | CT | Tryptophan | CD1 |  | CW |
| Threonine | CA |  | CT | Methionine | CG |  | CT | Tryptophan | CD2 |  | CW |
| Threonine | C |  | C | Methionine | SD |  | S | Tryptophan | NE1 |  | NC |
| Threonine | O |  | O | Methionine | CE |  | CT | Tryptophan | CE2 |  | CW |
| Threonine | CB |  | CT | Lysine | N |  | N | Tryptophan | CE3 |  | CA |
| Threonine | OG1 |  | OH | Lysine | CA |  | CT | Tryptophan | CZ2 |  | CA |
| Threonine | CG2 |  | CT | Lysine | C |  | C | Tryptophan | CZ3 |  | CA |
| Cysteine | N |  | N | Lysine | O |  | O | Tryptophan | CH2 |  | CA |
| Cysteine | CA |  | CT | Lysine | CB |  | CT |  | | | |
| Cysteine | C |  | C | Lysine | CG |  | CT |  | | | |
| Cysteine | O |  | O | Lysine | CD |  | CT |  | | | |
| Cysteine | CB |  | CT | Lysine | CE |  | CT |  | | | |
| Cysteine | SG |  | SH | Lysine | NZ |  | N3 |  | | | |
| Proline | N |  | N | Arginine | N |  | N |  | | | |
| Proline | CA |  | CT | Arginine | CA |  | CT |  | | | |
| Proline | C |  | C | Arginine | C |  | C |  | | | |
| Proline | O |  | O | Arginine | O |  | O |  | | | |
| Proline | CB |  | CT | Arginine | CB |  | CT |  | | | |
| Proline | CG |  | CT | Arginine | CG |  | CT |  | | | |
| Proline | CD |  | CT | Arginine | CD |  | CT |  | | | |
|  |  |  |  | Arginine | NE |  | N2 |  | | | |
|  |  |  |  | Arginine | CZ |  | CA |  | | | |
|  |  |  |  | Arginine | NH |  | N2 |  | | | |
