# Supplementary material for: On the Origins of Enzyme Inhibitor Selectivity and Promiscuity: A Case Study of Protein Kinase Binding to Staurosporine
Source: Chem Biol Drug Des. 2009 Jul;74(1):16–24. doi: 10.1111/j.1747-0285.2009.00832.x (PMC2737611; doi:10.1111/j.1747-0285.2009.00832.x)

**Appendix S2** Reference atoms for the classification of amino acids at the residue type level

21 residue types are included in the computer program; these are the 20 amino acids and water. Each amino acid residue is represented by a point at the position of its representative atom, usually located at a distinctive part near the end of the side chain as follow.


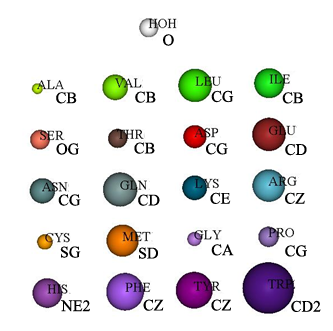

Supplement: Supplementary file 2 [file jpp0074-0016-SD2.doc]
