# Supplementary material for: On the Origins of Enzyme Inhibitor Selectivity and Promiscuity: A Case Study of Protein Kinase Binding to Staurosporine
Source: Chem Biol Drug Des. 2009 Jul;74(1):16–24. doi: 10.1111/j.1747-0285.2009.00832.x (PMC2737611; doi:10.1111/j.1747-0285.2009.00832.x)
Supplement: Supplementary file 3 [file jpp0074-0016-SD3.doc]

**Appendix S3** The shape-based dendogram constructed from 17 points in 17 residues, which are equivalent to the following residues in cAMP dependent protein kinase: LEU 49, GLY 50, VAL 57, ALA70, MET 71, LYS 72, VAL 104, MET 120, GLU 121, TYR 122, VAL 123, GLU 170, ASN 171, THR 183, ASP 184, GLU 127, LEU 173. This dendrogram places the same type of kinase in different complexes in the same branch regardless of the bound ligand. The staurosporine binding structures are clustered into one half of the tree
