# Supplementary material for: On the Origins of Enzyme Inhibitor Selectivity and Promiscuity: A Case Study of Protein Kinase Binding to Staurosporine
Source: Chem Biol Drug Des. 2009 Jul;74(1):16–24. doi: 10.1111/j.1747-0285.2009.00832.x (PMC2737611; doi:10.1111/j.1747-0285.2009.00832.x)

**Appendix S4** Distances between pairs of 15 frequently occurring residues chosen for Multiple Linear Regression. **These distances are measured from** the centres of the distinctive parts of residues that are equivalent to these residues in cAMP dependent protein kinase (PKA).

Point 1: LEU 49 Point 2: GLY 50

Point 3: VAL 57 Point 4: ALA70

Point 5: MET 71 Point 6: LYS 72

Point 7: VAL 104 Point 8: MET 120

Point 9: GLU 121 Point 10: TYR 122

Point 11: VAL 123 Point 12: GLU 170

Point 13: ASN 171 Point 14: THR 183

Point 15: ASP 184


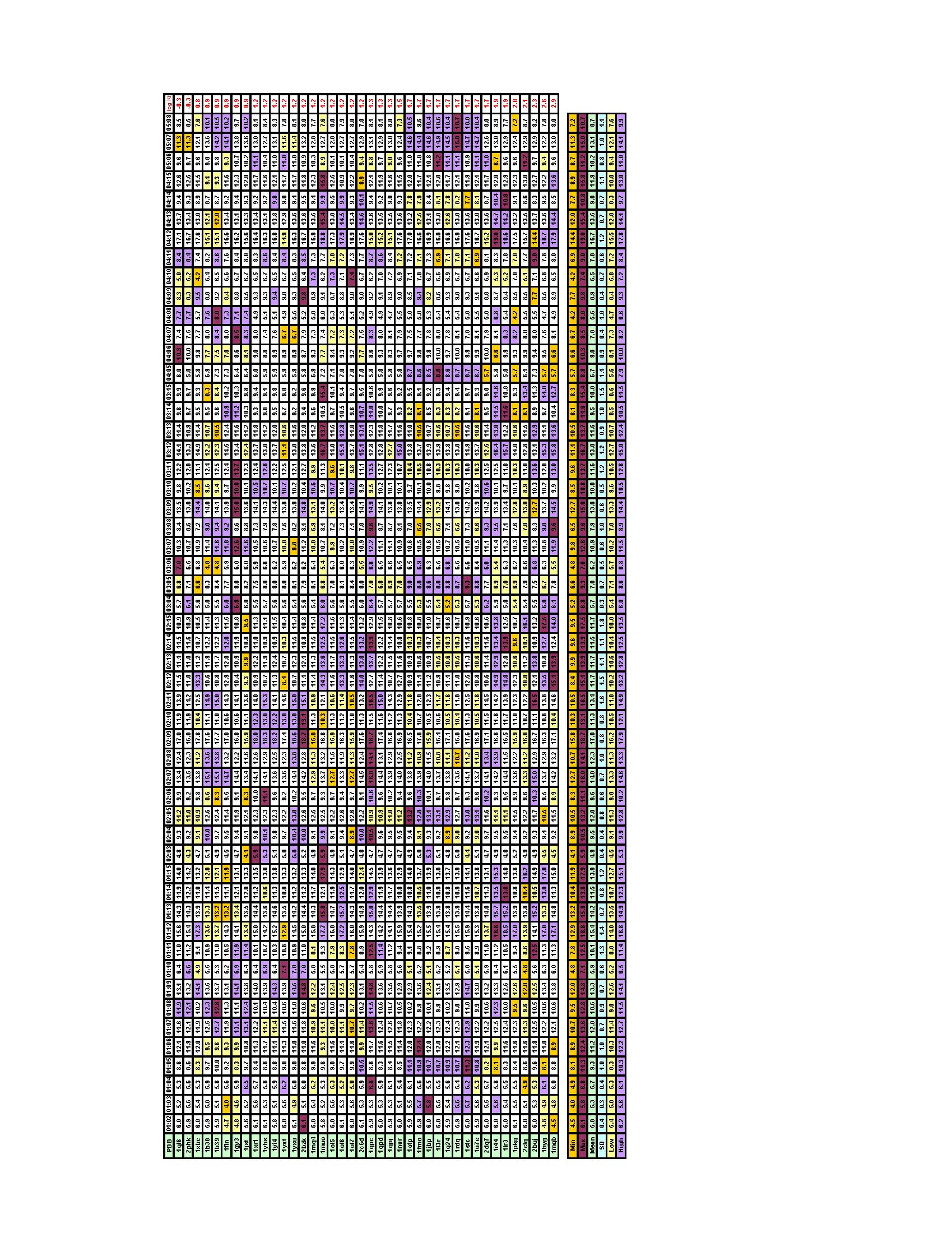


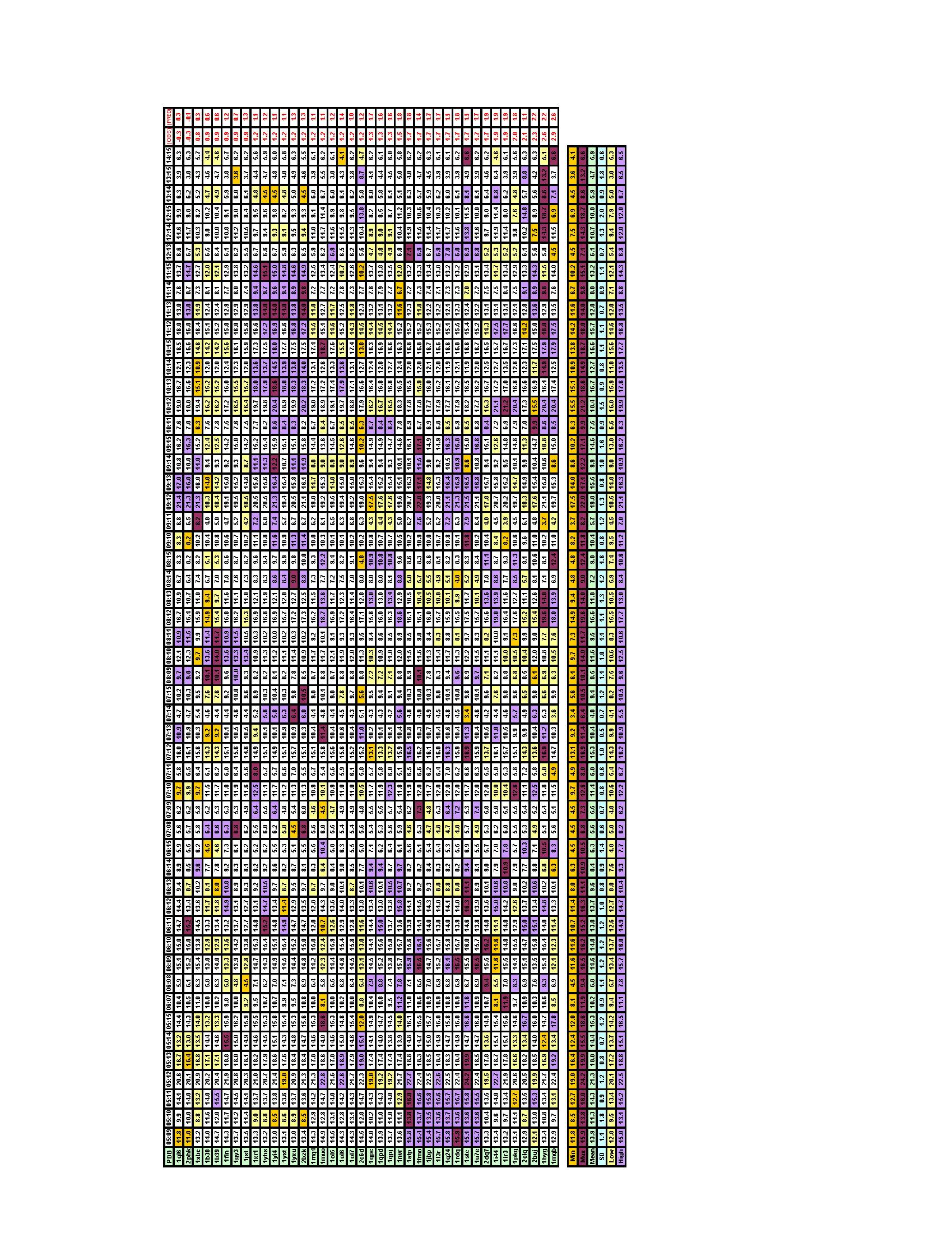

Supplement: Supplementary file 4 [file jpp0074-0016-SD4.doc]
