# Supplementary material for: On the Origins of Enzyme Inhibitor Selectivity and Promiscuity: A Case Study of Protein Kinase Binding to Staurosporine
Source: Chem Biol Drug Des. 2009 Jul;74(1):16–24. doi: 10.1111/j.1747-0285.2009.00832.x (PMC2737611; doi:10.1111/j.1747-0285.2009.00832.x)
Supplement: Supplementary file 5 [file jpp0074-0016-SD5.doc]

**Appendix S5**

The predictive power of the multiple linear regression equations, tested by leaving out randomly selected test sets, shows R^2^ equal to 0.75 and 0.71 respectively
